# Use of Mitomycin C to reduce the incidence of encapsulated cysts following ahmed glaucoma valve implantation in refractory glaucoma patients: a new technique

**DOI:** 10.1186/1471-2415-14-107

**Published:** 2014-09-06

**Authors:** Minwen Zhou, Wei Wang, Wenbin Huang, Xiulan Zhang

**Affiliations:** Zhongshan Ophthalmic Center, State Key Laboratory of Ophthalmology, Sun Yat-Sen University, 54S.Xianlie Road, Guangzhou, 510060 China; Department of Ophthalmology, Shanghai First People’s Hospital, School of Medicine, Shanghai JiaoTong University, Shanghai, China; Shanghai Key Laboratory of Fundus Disease, Shanghai, China

**Keywords:** Refractory glaucoma, Ahmed glaucoma valve implantation, Encapsulated cyst, Mitomycin C

## Abstract

**Background:**

To evaluate the surgical outcome of Ahmed glaucoma valve (AGV) implantation with a new technique of mitomycin C (MMC) application.

**Methods:**

This is a retrospective study. All patients with refractory glaucoma underwent FP-7 AGV implantation. Two methods of MMC application were used. In the traditional technique, 6 × 4 mm cotton soaked with MMC (0.25–0.33 mg/ml) was placed in the implantation area for 2–5mins; in the new technique, the valve plate first was encompassed with a thin layer of cotton soaked with MMC, then inserted into the same area. A 200 ml balanced salt solution was applied for irrigation of MMC. The surgical success rate, intraocular pressure (IOP), number of anti-glaucoma medications used, and postoperative complications were analyzed between the groups.

**Results:**

The surgical outcomes of two MMC applied techniques were compared. The new technique group had only one case (2.6%) of encapsulated cyst formation out of 38 eyes, while there were eight (19.5%) cases out of 41 eyes the in traditional group. The difference was statistically significant (*P* = 0.030). According to the definition of success rate, there was 89.5% in the new technique group and 70.7% in the traditional group at the follow-up end point. There was a significant difference between the two groups (*P* = 0.035). Mean IOP in the new technique group were significantly lower than those of the traditional group at 3 and 6 months (*P* < 0.05).

**Conclusions:**

By using a thin layer of cotton soaked with MMC to encompass the valve plate, the new MMC application technique could greatly decrease the incidence of encapsulated cyst and increase the success rate following AGV implantation.

## Background

Ahmed glaucoma valve (AGV) implantation has been widely used and has been proved to be an effective method for treating refractory glaucoma [1–4]. Several studies in the literature have reported success rates of AGV implantation ranging from 49% to 83.6% [1, 3, 5–7]. Encapsulated cyst formation is one of the main reasons for failure [8]. The proliferation of fibrous tissue around the implant plates blocks the diffusion of aqueous humor and elevates intraocular pressure (IOP) [9]. Adjunctive use of antimetabolites can greatly inhibit fibrosis [10, 11], and mitomycin C (MMC) has been used extensively in filtering and glaucoma drainage device implant surgery [12, 13]. However, how to use MMC more effective has remained to be explored further. Heuer et al. [14]. Found that double-plate Molteno implantation more frequently affords IOP control than single-plate Molteno. Assuming that the expanded surface area of the implant plate allows reduced occurrence of encapsulated cyst, it is also supposed that expanding the MMC function area in the scleral bed where the AGV is placed may decrease encapsulated cyst formation. Unfortunately, the cotton soaked with MMC and inserted into the implantation area often rolls into a mass, without a guarantee of enough size. Therefore, we improved the method by introducing a novel way for MMC to be used: the valve plate was first encompassed with a thin layer of cotton soaked with MMC, then insert into the implanted area. In this study, we evaluated its surgical outcomes to see whether the new method could produce better surgical results.

## Methods

### Patients and inclusion criteria

This was a retrospective study of patients diagnosed with refractory glaucomas (including failed filtration, uveitic glaucoma, pseudophakia, and traumatic glaucoma) who underwent AGV implantation at the Glaucoma Department of Zhongshan Ophthalmic Center. Consecutive patients followed up at the Zhongshan Ophthalmic Center from October 2008 and January 2013 were included in this study. From October 2008 to January 2010, we employed the traditional method in our hospital, and we converted to the new technique from January 2010. It was approved by the Ethical Review Committee of Zhongshan Ophthalmic Center and adhered to the provisions of the Declaration of Helsinki for research involving human subjects.

Best corrected visual acuity (BCVA), IOP, number of antiglaucoma medications, and systemic diseases were examined by chart review. Demographic data, such as age, sex, prior surgery history, and subtypes of glaucoma were collected. All patients received MMC application during the surgery. Age less than 18 years, previous aqueous shunt surgery in the same eye, prior scleral buckling procedures, and without MMC application, were all factors for excluding patients from the study.

### Surgical techniques and MMC application

**Figure 1:**
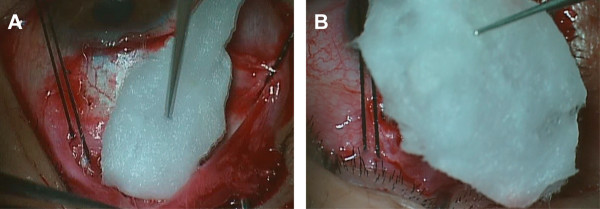
**The different way of MMC application technique. A**: The traditional MMC application way: a piece of cotton soaked MMC was inserted directly. **B**: The new technique way: a thin layer of cotton soaked with MMC to encompass the valve plate, then inserted the place of AGV placed.

One glaucoma specialist (XZ) performed all the FP-7 AGV implantation surgeries, using the same techniques. A fornix-based flap of the conjunctiva and Tenon capsule was created in the superior temporal quadrant. However, in the patients who had undergone previous eye surgery, such as trabeculectomy, causing scarring of the conjunctiva of the superior temporal quadrant, we used an inferior temporal quadrant incision. The tube of the AGV was flushed with a balanced salt solution through a scleral track to ensure patency before insertion. In order to decrease the possibility of overfiltering following AGV implantation, the tube was ligated tightly to restrict aqueous flow, using 8–0 polyglactin absorbable sutures, in all patients. The AGV was positioned in the middle of the quadrant, with the anterior edge of the plate 10 mm or more posterior to the superior temporal corneoscleral limbus. Before the AGV was placed, MMC (0.25–0.33 mg/ml, 2–5 min) was applied in all patients (Figure 1). The concentration and time of MMC depended on the judgment of the risk of failure of the surgery by the surgeon. In the traditional manner, a piece of cotton (6 × 4 mm) soaked with MMC was placed in the appointed area. However, the wet cotton often rolled into a mass, without a guarantee of enough size. In the improved manner, the valve plate was first encompassed with a thin layer of cotton soaked with MMC, then inserted into the same area. After 2–5 minutes, the cotton pieces and the encompassed AGVs were removed and irrigated with 200 ml of balanced salt solution. Then, the valve plate was sutured to the sclera with 6–0 nylon sutures through the anterior positional holes of the body of the valve plate. A half-thickness, rectangular, 4 × 6 mm, limbal-based scleral flap was created. The tip of the drainage tube was then cut and beveled upwards, in order to extend it by 2 mm into the anterior chamber. Paracentesis in the inferior temporal peripheral cornea was performed, and viscoelastic was injected to maintain the anterior chamber before tube insertion. A 23-gauge needle punctured the anterior chamber under the scleral flap, and the drainage tube was inserted. The tube was sutured to the episcleral surface with 8–0 polyglactin sutures. The scleral flap over the drainage tube was reattached to the sclera and sutured with 10–0 nylon sutures. The conjunctiva and Tenon capsule were reapproximated to the limbus with 8–0 polyglactin sutures. Topical prednisolone acetate 1% (prednisolone acetate ophthalmic suspension, USP; PA) was administered four times daily for four weeks, and was then replaced with non-steroid anti-inflammatory drug eye drops (pranoprofen 0.1% [Senju, Japan; PF]) for two weeks. Glaucoma medications were prescribed when the postoperative IOP was greater than 21 mm Hg, and the medications were added or removed according to the IOP level. Topical β-blockers were the first line of therapy. Topical carbonic anhydrase inhibitor and topical α2-adrenergic agonists were added as a second line of therapy. Systemic medications to decrease IOP were applied if necessary. When the IOP was reduced after treatment, antiglaucoma agents were gradually withdrawn during the follow-up.

### Postoperative follow-up

Postoperative IOP and VA were recorded at each visit after AGV implantation surgery. The number of postoperative glaucoma medications and postoperative complications were recorded. The postoperative visits were performed after 1 day, 1 week, 1 month, 3 months, and every 6 months thereafter.

### Evaluation criteria

Postoperative survival was defined as IOP <21 mm Hg, with or without glaucoma medications, and without significant visually threatening complications (endophthalmitis, retinal detachment, suprachoroidal hemorrhage, preseptal cellulitis, or persistent hypotony [IOP < 5 mm Hg]). Failure was defined as IOP not less than 21 mm Hg, IOP < 5 mm Hg on two consecutive follow-up visits after three months, or loss of light perception, or the need for further surgery or laser to control IOP [15].

### Statistical analysis

The data were processed and statistically analyzed using SPSS for Windows XP (Version 13.0; SPSS, Chicago, IL). The Mann–Whitney *U* test was used for variables with a skewed distribution, and the chi-square or Fisher’s exact test was used for categorical variables. An independent sample *t* test was used to compare normally distributed continuous variables data between the two groups. To compare the IOPs and glaucoma medications at various time points before and after operation, the Wilcoxon signed-rank test was used. Success rates in both groups were compared using Kaplan–Meier survival curves and the log rank test. *P* values of <0.05 were considered statistically significant.

## Results

Seventy-nine eyes of 79 patients who fulfilled the inclusion criteria were included in the study. MMC applied with the traditional technique was performed in 41 eyes (traditional group), while the new technique was performed in the remaining 38 eyes (new technique group). The minimum required follow-up period after surgery was six months. Mean follow-up times were 19.89 ± 8.29 months for the new technique group and 18.10 ± 8.71 months for the traditional group (*P* = 0.938). The demographic and preoperative data of the two groups are presented in Table 1. There were no significant differences in sex, mean age, mean IOP, mean BCVA, mean glaucoma medication, or number of previous glaucoma surgeries between the two groups.

**Table 1:** **Demographic and preoperative data of different group patients**

	New technique (n = 38)	Traditional (n = 41)	***P***
Age (y), mean ± SD	42.34 ± 13.69	38.29 ± 15.32	0.220^a^
Sex			0.150^b^
Male, n (%)	23 (60.5)	31 (75.6)	
Female, n (%)	15 (39.5)	10 (24.4)	
Mean IOP (mm Hg), mean ± SD	41.97 ± 10.58	43.15 ± 9.63	0.608^a^
Mean glaucoma medication,mean ± SD	3.13 ± 0.58	3.24 ± 0.92	0.460^c^
Mean follow-up time (month), mean ± SD	19.89 ± 8.29	18.10 ± 8.71	0.938^a^
Mean best corrected visual acuity (logMAR)	1.82 ± 1.25	2.24 ± 1.11	0.107^c^
Mean MMC concentration (mg/ml)	0.29 ± 0.04	0.28 ± 0.04	0.155^a^
Mean MMC duration (min)	3.24 ± 0.97	3.00 ± 1.02	0.296^a^
Previous glaucoma surgeries history, n (%)	17 (44.7)	19 (46.3)	0.886^b^
Diagnosis			0.992^b^
Uveitic glaucoma, n (%)	10 (26.3)	11 (26.8)	
NVG, n (%)	13 (34.2)	15 (36.6)	
Traumatic glaucoma, n (%)	4 (10.5)	3 (7.3)	
ICE syndrome, n (%)	2 (5.3)	2 (4.9)	
Failed trabeculectomy, n (%)	9 (23.7)	10 (24.4)	

^a^independent sample *t* test.

^b^chi-square test.

^c^Mann–Whitney *U* test.

*Abbreviations:* *SD* indicates standard deviation, *IOP* introcular pressure, *MMC* mitomycin C, *NVG* neovascular glaucoma, *ICE* Irido-corneal endothelial.

Compared with preoperative IOP, the two groups showed a statistically significant IOP decrease at all follow-up intervals (*P* < 0.05, Wilcoxon signed-rank test). IOP was lower in the traditional group than in the new technique group in postoperative day 1 and week 1. Nevertheless, the new technique group showed lower IOPs thereafter up to the end of the study in postoperative month 30. The new technique group, compared with traditional group, showed significantly lower IOP at month 3 (*P* = 0.029) and month 6 (*P* = 0.043). Table 2 display the mean IOPs at all time intervals in both groups.

**Table 2:** **Mean IOP and mean glaucoma medications required in both groups at all follow-up time intervals (mean ± SD)**

Follow-up time	New technique (n = 38, mm Hg)	Traditional (n = 41, mm Hg)	P*
Preoperative			
IOP (mmHg)	41.97 ± 10.58	43.15 ± 9.63	0.608
Glaucoma medications	3.13 ± 0.58	3.24 ± 0.92	0.460
Postoperative 1 day			
IOP (mmHg)	20.03 ± 9.04	16.15 ± 8.66	0.055
Glaucoma medications	0.21 ± 0.66	0.22 ± 0.00	0.683
Postoperative 1 week			
IOP (mmHg)	12.37 ± 5.05	11.46 ± 4.06	0.381
Glaucoma medications	0.11 ± 0.51	0.00 ± 0.00	0.505
Postoperative 1 month			
IOP (mmHg)	13.45 ± 3.50	15.15 ± 6.04	0.134
Glaucoma medications	0.03 ± 0.16	0.17 ± 0.38	0.035
Postoperative 3 months			
IOP (mmHg)	14.26 ± 4.96	17.34 ± 7.09	0.029
Glaucoma medications	0.13 ± 0.53	0.59 ± 1.09	0.025
Postoperative 6 months			
IOP (mmHg)	14.71 ± 3.01	17.27 ± 7.10	0.043
Glaucoma medications	0.34 ± 0.63	0.71 ± 1.17	0.442
Postoperative 12 months			
IOP (mmHg)	15.61 ± 5.20	16.90 ± 5.97	0.384
Glaucoma medications	0.67 ± 1.03	0.63 ± 1.16	0.590
Postoperative 18 months			
IOP (mmHg)	16.23 ± 5.13	16.38 ± 4.39	0.916
Glaucoma medications	0.85 ± 1.16	0.50 ± 1.00	0.310
Postoperative 24 months			
IOP (mmHg)	15.85 ± 1.96	17.33 ± 3.83	0.207
Glaucoma medications	0.79 ± 0.97	0.64 ± 1.15	0.667
Postoperative 30 months			
IOP (mmHg)	15.75 ± 2.05	16.20 ± 3.90	0.788
Glaucoma medications	0.63 ± 0.92	0.60 ± 1.34	0.833

*Independent sample *t*-test or Mann–Whitney *U* test.

*Abbreviations:* *IOP* introcular pressure, *SD* standard deviation.

Table 2 compares the mean numbers of antiglaucoma medications required in both groups at all time intervals. Medication use for both groups after surgery was significantly decreased at all follow-up time points when compared with preoperative values (*P* < 0.05, Wilcoxon signed-rank test). There were no statistically significant differences between the groups at most of time point in terms of the mean number of medications. However, the new technique group had a significantly lower mean number of medications than the traditional group at the month 1 (*P* = 0.035) and month 3 (*P* = 0.025) postoperative follow-up visit.

**Figure 2:**
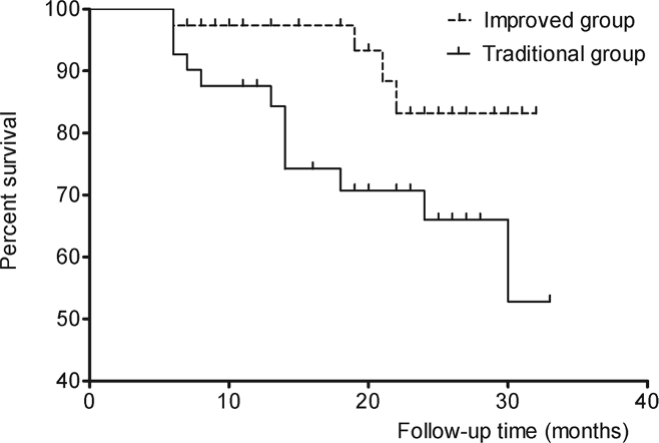
**Cumulative survival curves showed new technique group had a greater survival than traditional group after AVG implantation.** There was significant difference between the 2 groups (*P* = 0.035).

Kaplan–Meier survival analysis showed that the success rates for the new technique and traditional groups were 97.4% and 87.8% at 12 months, respectively, and 89.5% and 73.2% at 24 months, respectively. The success rate of the new technique group was significantly higher than that of the traditional group (*P* = 0.035, log rank test) (Figure 2). After the endpoint of follow-up, failure had occurred in 4 patients (10.5%) in the new technique group and 11 patients (26.8%) in the traditional group. Table 3 showed the reasons for failure in both groups.

**Table 3:** **Reasons for failure in both groups**

	New technique (n = 38)	Traditional (n = 41)
High IOP* (>21 mmHg)	2 (5.3%)	9 (22.0%)
Low IOP* (<5 mmHg)	1 (2.6%)	0
Progression to NLP	1 (2.6%)	1(2.4%)
Additional glaucoma surgery	0	1(2.4%)

*IOP-related failures require 2 consecutive visits at or after 3 months in which the criterion is not met.

During the follow-up period, visual acuity remained unchanged relative to pre-operative values. There were no significant differences in visual acuity between the 2 groups at all time points (Table 4).

**Table 4:** **Mean best corrected visual acuity (logMAR) in both groups at all follow-up time intervals (mean ± SD)**

Follow-up time	New technique (n = 38, mm Hg)	Traditional (n = 41, mm Hg)	P*
Preoperative	1.82 ± 1.25	2.24 ± 1.11	0.107
Postoperative 1 day	1.89 ± 1.22	2.30 ± 1.09	0.124
Postoperative 1 week	1.87 ± 1.21	2.18 ± 1.18	0.225
Postoperative 1 month	1.79 ± 1.25	2.12 ± 1.17	0.177
Postoperative 3 months	1.79 ± 1.25	2.10 ± 1.19	0.228
Postoperative 6 months	1.91 ± 1.35	2.09 ± 1.20	0.386
Postoperative 12 months	2.04 ± 1.21	2.11 ± 1.26	0.745
Postoperative 18 months	2.06 ± 1.24	2.02 ± 1.26	0.991
Postoperative 24 months	1.86 ± 0.91	2.28 ± 1.25	0.398
Postoperative 30 months	1.77 ± 0.92	2.62 ± 0.86	0.141

*Mann–Whitney *U* test.

*Abbreviations:* *IOP* introcular pressure, *SD* standard deviation.

As shown in Table 5, postoperative complications included encapsulated cyst formation, choroidal effusion, flat anterior chamber, hypotony maculopathy, and hyphema. The most common complication in the eyes of the traditional group was encapsulated cyst formation, with incidences in eight eyes (19.5%), while there was only an incidence in one eye (2.6%) in the new technique group. Statistically significant differences were detected between the two groups when comparing encapsulated cyst formation complications (*P* = 0.030). Flat anterior chamber occurred in five eyes (13.2%) in the new technique group. There were no statistically significant differences in incidences of other postoperative complications between the groups.

**Table 5:** **Postoperative complications in both groups**

Complications	New technique (n = 38)	Traditional (n = 41)	***P***
Encapsulated cyst formation, n (%)	1 (2.6%)	8 (19.5%)	0.030*
Choroidal effusion, n (%)	3 (7.9%)	1 (2.4%)	0.612
Flat anterior chamber, n (%)	5 (13.2%)	1 (2.4%)	0.072
Hypotony maculopathy, n (%)	1 (2.6%)	0 (0%)	0.481
Hyphema, n (%)	2 (5.3%)	4 (9.8%)	0.676

Fisher’s exact test.

*P < 0.05 between the two groups.

## Discussion

AGV implantation allows aqueous drainage via a tube inserted into the anterior chamber to a posterior plate sutured to the episclera. The aqueous humor crosses the surrounding bleb wall by passive diffusion, and it is removed from the periocular space by venous capillaries or lymphatics [16, 17]. However, when proliferation of fibrous tissue around the plate forms, it restricts aqueous humor diffusion through the capsule, followed by a gradual elevation of IOP, and then, encapsulated cyst formation [8]. Encapsulated cyst formation is the most frequent reason for glaucoma drainage device implant surgery failure [8]. Adjunctive use of MMC is still controversial; while most studies have concluded that adjunctive use of MMC is beneficial for improving success rates [11, 18, 19], other studies have found that MMC did not increase the short- or intermediate-term success rates of AGV implantation [20, 21]. Thus, further study is expected to reveal whether adjunctive use of MMC is beneficial, as well as how to use it more effectively in AGV implantation.

In the process of AGV implantation, the traditional method for placing MMC is to take a piece of cotton or sponge soaked with MMC into the middle of the quadrant where the valve was to be implanted. In fact, the cotton or sponge often folds or rolls into a mass at the scleral bed, limiting the anti-fibrotic function of MMC. In this study, the new technique, which overcame this shortcoming, was able to guarantee enough fixed space for MMC to function. Therefore, the novel technique could greatly decrease encapsulated cyst incidences and significantly increase surgical outcomes.

This is the first study to compare the surgical outcome and complication rates associated with the use of MMC in AGV implantation, using the traditional and new methods. Both methods showed efficacy and safety during AGV implantation, and they showed a similar trend in postoperative IOP control and the use of glaucoma medication. Kaplan–Meier survival curves showed statistically significant differences between the groups, which might be a result of the lower incidences of encapsulated cyst formation in the new technique group (one eye, 2.6% vs. eight eyes, 19.5% in the traditional group).

Comparing our findings with other reported series is problematic, as some authors do not consider the formation of an encapsulated cyst as a complication and, therefore, do not report it [22–24]. However, other studies have reported differences in incidences of encapsulated cyst. Lai [25], in a series of 65 eyes undergoing AGV implantation, reported that 16 eyes (24.6%) developed encapsulated cyst as a postoperative complication. Similarly, a prospective, comparative study showed that five eyes (14.7%) had incidences of encapsulated cyst after AGV implantation [6]. In short, encapsulated cyst formation is often referred to as a late complication after glaucoma implant insertion in adults, with an appearance varying from 5% to 30%, depending on study design, follow-up time, and patient selection. In our study, encapsulated cyst occurred in only 2.6% of the new technique group. Therefore, by using a thin layer of cotton soaked with MMC to encompass the valve plate, this novel MMC application technique could greatly decrease the incidence of encapsulated cyst.

On the other hand, the incidence of flat anterior chamber using the new technique method was higher than using the traditional method. In fact, to avoid postoperative hypotony, the tube was ligatured tightly, using 8–0 polyglactin suture, to restrict aqueous flow during the surgery. Therefore, the use of adjunctive MMC may be another cause of hypotony, besides leakage around the tube, a decrease in aqueous production, and overfiltration [26]. Whether the new technique method of MMC application allows more range for MMC functioning to cause more flat chamber incidence needs to be investigated further.

The main limitation of this study is the nonrandomized design. We took every possible step to reduce potential bias, and the final data were subjected to careful statistical analysis. The crucial criterion for any “randomization” is to have groups at the baseline comparable in demographic and clinical characteristics. In this study, that was the case. The second limitation is that different MMC concentrations and times in different patient might affect result of study.

## Conclusions

In conclusion, this study indicates that the new technique for MMC application may provide a better chance for patients to decrease the incidence of encapsulated cyst, when compared with the traditional method. In addition, there was a tendency for lower IOP and higher complete success rate in the new technique group.
